# Therapeutic Management of a Substantial Pelvic Aneurysmatic Bone Cyst Including the Off-Label Use of Denosumab in a 35-Year-Old Female Patient

**DOI:** 10.1155/2017/9125493

**Published:** 2017-10-17

**Authors:** D. Ntalos, M. Priemel, C. Schlickewei, D. M. Thiesen, J. M. Rueger, A. S. Spiro

**Affiliations:** ^1^Department of Trauma, Hand, and Reconstructive Surgery, University Medical Center Hamburg-Eppendorf, Hamburg, Germany; ^2^Department of Pediatric Orthopedic Surgery, Children's Hospital Hamburg-Altona, Hamburg, Germany

## Abstract

Aneurysmal bone cysts (ABC) are benign bone tumors, which are highly vascularized. The main course of treatment is curettage followed by bone grafting or cement insertion. Still recurrence remains a main problem for patients. Denosumab is a monoclonal antibody, which acts as an inhibitor of the RANK/RANKL pathway, diminishing bone turnover. Recent case reports have shown that Denosumab can be a promising therapeutic agent for people suffering from therapy-resistant ABC. We report the case of a 35-year-old female patient presenting with a pronounced ABC of the pelvis. Since the tumor was inoperable, Denosumab was administered, leading to a significant shrinkage of the lesion, which allowed surgical intervention. Upon recurrence, Denosumab was restarted putting the patient once more into remission. Follow-up was four years overall with a clinical and radiological stable disease for fifteen months after final discontinuation of the monoclonal antibody. Therefore, our case further underlines the potential of Denosumab in the treatment of ABC.

## 1. Introduction

Aneurysmal bone cysts (ABC) were first described in 1942 by Jaffe and Lichtenstein and account for almost one per cent of all benign bone tumors [1, 2]. Typically, the tumor occurs in the first two decades of a person's life, though up to twenty per cent are diagnosed later on [3]. ABC are highly vascular, eccentric, osteolytic lesions of unknown origin which most commonly manifest themselves in the metaphysis of the long bones, especially the femur and tibia [4, 5]. Involvement of the pelvis is rather rare and occurs, depending on the study, in four to twelve per cent of the cases [2, 4, 5]. The pathogenesis of these lesions, although having been known now for more than seventy years, still remains unclear. Among vascular and traumatic factors, recent studies describe genetic alterations as well [3, 6, 7]. Treatment options for aneurysmal bone cysts comprise surgical resection or curettage followed by bone grafting or cement insertion as well as arterial embolization or direct injection of the cyst with sclerosing agents such as polidocanol or local injection of doxycycline [8–10]. Focusing on the pelvis, optimal treatment is challenging since the lesions are usually very large, highly vascularized and the anatomical relation to the acetabulum and neurovascular structures reduces the chance of adequate resection [2, 11, 12].

As a possible new therapeutic agent for ABC, the use of Denosumab has been described to our knowledge in only five cases so far. Denosumab operates as an inhibitor of RANKL, which is known to increase bone turnover due to stimulating osteoclastic cells. The monoclonal antibody has therefore been implemented in the treatment of osteoporosis, skeletal metastases, and giant cell tumors of the bone with good results. In context of aneurysmal bone cysts, there is still a lack of data. The five existing cases show very promising results for Denosumab being a treatment option for ABC though, which is in accordance with our findings [12–16].

## 2. Case Report

**Figure 1:**
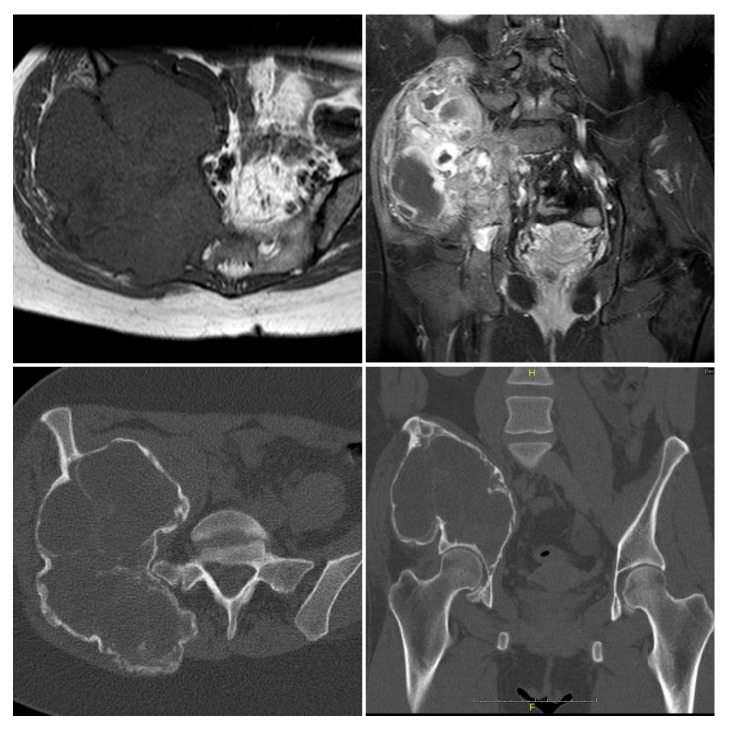
Initial MR and CT Imaging showing a substantial right-sided cystic tumor of the pelvis infiltrating associated neuroforamina.

A 35-year-old female patient presented herself as an outpatient in 2012 with a two-week history of pain in the right leg and hyposensibility of the right calf. For further investigation, an MRI of the lumbar spine was performed showing a right-sided cystic tumor of the pelvis instead of a suspected disc herniation. In the following, an abdominal CT scan was performed revealing an osteolytic involvement of the right ileum, os sacrum, and the right-sided neuroforamina s1–s4 (Figure 1).

On physical examination, the patient showed a moderate swelling of the right gluteal region with a slight reduction in range of motion of the right hip due to severe pain during leg movements. Otherwise the exam was unremarkable with normal blood circulation in both legs and intact sensitivity except for the right dorsal calf.

To further evaluate the tumor, an MRI scan of the pelvis and an open biopsy were performed (Figure 1).

**Figure 2:**
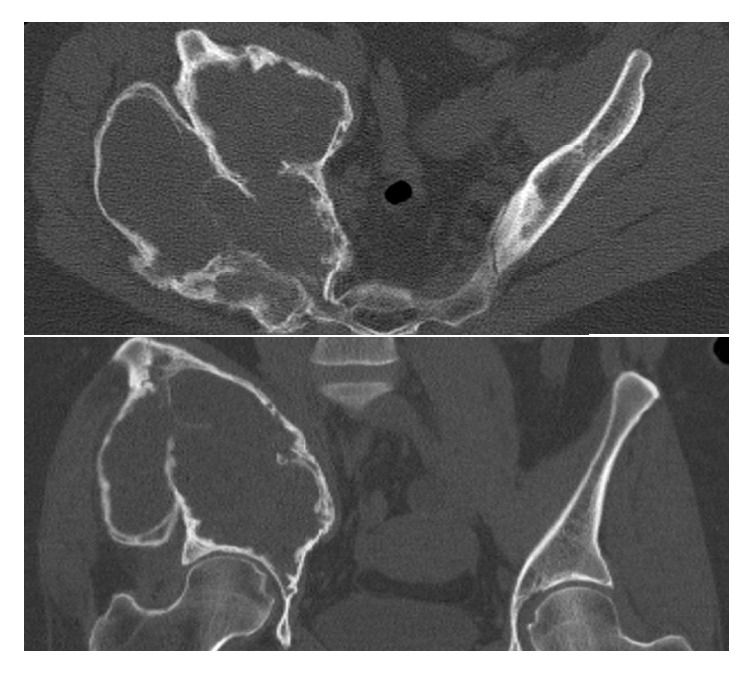
Preoperative follow-up CT scan showing a notable increase in cortical thickness.

The suspected diagnosis of an aneurysmal bone cyst was confirmed by histopathological examination. Treatment options were discussed in an interdisciplinary tumor board. Surgical intervention was not recommended due to inaccessibility. Therefore, selective arterial embolization was performed by our radiologists. Additionally, the off-label use of 60 mg of Denosumab, once every four weeks, was initiated. It was decided to start the patient with 60 mg and not 120 mg to assess a potential therapeutic effect while keeping side-effects low. Due to the unstable situation of the right pelvis, the patient was advised to use crutches with no weight bearing of the right leg. Follow-up examination and CT imaging were performed every three months and revealed a significant reduction in pain and a notable increase in cortical thickness, while less mineralization of the cyst cavity was seen (Figure 2). Therefore, Denosumab dosages were not changed. Additionally, an increase to partial weight bearing of the leg up to 20 kg was possible and therapy with Denosumab was discontinued after a total course of twelve months. During this time no remarkable side-effects were detected. During interdisciplinary discussions, surgical treatment via curettage and a combination of bone grafting and cement implantation of the defect was planned. Prior to surgery embolization of the remaining arteries was performed again.

Surgery included curettage, implantation of autologous spongiosa, alloplastic bone grafts, artificial bone material, and bone cement.

A follow-up CT scan six months after surgery revealed a significant tumor progress with decreased cortical thickness. Since there was a good response to Denosumab before, the treatment was restarted.

**Figure 3:**
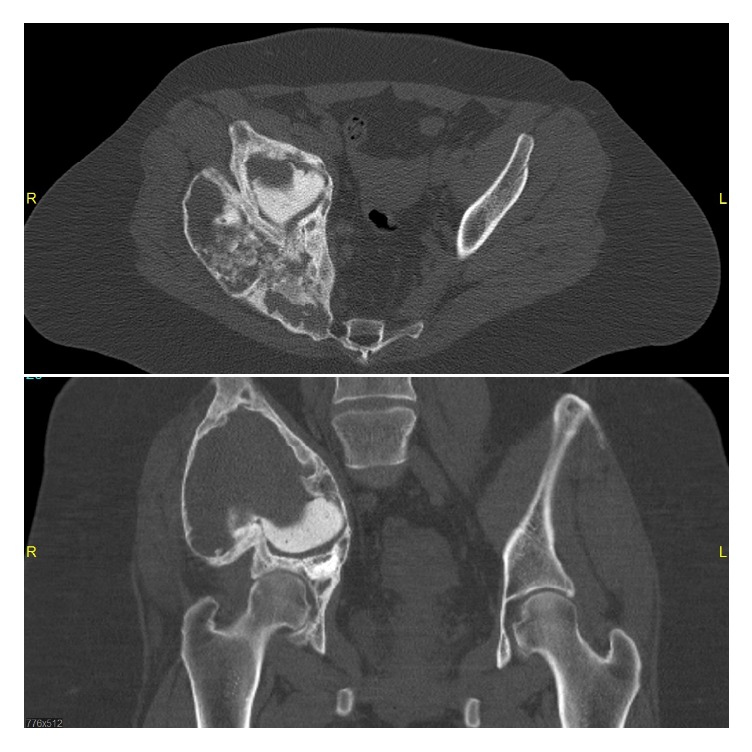
Follow-up CT scan of the pelvis showing stable disease after discontinuation of the second cycle of Denosumab treatment for fifteen months.

Follow-up imaging showed a significant response with tumor reduction, recalcification, and increasing stability of the pelvis again. Thus Denosumab therapy was continued, controlling the aforementioned lesion and allowing the patient to fully bear weight on the said leg. In the following, laboratory tests revealed signs of low turnover osteoporosis with low calcium, phosphate, and 25-hydroxyvitamin D3 levels combined with increased parathyroid hormone (PTH) levels resulting in discontinuation of Denosumab after a total of 17 months during the second cycle. Until today the overall patient follow-up is about four years with clinical examinations being performed in our outpatient clinic every 6–12 months. Denosumab has been discontinued for fifteen months with clinical and radiological examination showing a stable disease so far (Figure 3).

## 3. Discussion

There are several treatment options for aneurysmal bone cysts. Surgical treatment includes en bloc resection or curettage combined with bone grafting or cement application [2, 11, 12, 17]. Either one can be combined with neoadjuvant or adjuvant treatment. Additionally, nonsurgical management using cryotherapy, sclerotherapy, doxycycline injections, radionuclide ablation, arterial embolization, or radiotherapy has been described to yield good results as well. Despite these various options recurrence of ABC remains a main problem [2, 11, 12, 17].

Resecting the tumor with wide margins has been described to have the lowest rate of recurrence but due to anatomical reasons it is rarely possible, as can be seen in our case. In this context, the effect of Denosumab can be used to diminish tumor size and enhance operability of the lesion. However, increasing bone mineralization and development of septal bone separations caused by Denosumab can complicate surgical treatment.

In our case, the patient relapsed within six months after surgical curettage and graft insertion. Data ascertained by Mankin et al. show recurrence rates up to 30% after curettage alone and close to 25% following curettage succeeded by allograft or cement implantation. Based on different risk factors such as younger age, aggressiveness of the lesion, and type of treatment, rates can be even higher leading up to 59% [18, 19]. Therefore, the course of disease as displayed by our patient is quite common and calls for potential long-term intervention.

Lange et al. and Pauli et al. have shown that recurrent ABC could be well controlled by Denosumab application [12, 14]. Our case supports these findings and in particular the use of Denosumab in inoperable and recurrent ABC as a promising alternative. Since we used a multimodal approach including repeated embolization, the treatment results may have been caused in part by embolization and not completely by Denosumab.

The underlying mechanism of the monoclonal antibody in context of ABC remains unclear. Denosumab acts as an inhibitor within the RANKL/RANK (receptor activator of NF-*κ*B ligand/receptor activator of NF-*κ*B) pathway. Since RANKL works as an essential differentiation factor for osteoclasts, it is a widely accepted theory that bone resorption is stimulated by this pathway and therefore could be decreased by the use of Denosumab [13, 20, 21]. Clinical data has already shown the successful therapeutic use of Denosumab in the context of osteoporosis, bone metastases, and recently giant cell tumor, which share immunohistochemical attributes with ABC. Furthermore, recent studies suggested RANKL to play an important part in ABC as well which could explain Denosumab's effects on ABC [15, 22–24].

Treating ABC with Denosumab is thus not only theoretically astute but has shown promising results in a very small group of patients, with five reported cases so far [12–16].

Furthermore Denosumab was suggested to potentially be used as first-line treatment as shown by the case of a five-year-old boy responding very well to the singular use of the antibody [15].

Upon considering Denosumab as a treatment option, one has to take notice of the side-effects. Even though Denosumab seems to be a well-tolerated drug, especially when facing long-term treatment several side-effects are described, with the most noteworthy seeming to be osteonecrosis of the jaw (ONJ) [25–27]. One study has shown an occurrence rate of 5% of ONJ in patient undergoing treatment [25]. These numbers though were derived in a population taking twice the amount that our patient received and ONJ was not observed in other trials where lower dosages of Denosumab were administered [26–28]. Furthermore Denosumab has been associated with severe hypocalcemia, atypical femur fractures, and rebound bone resorption phenomena [29]. Therefore, considering that there are alternative treatment options such as polidocanol [30] and doxycycline injection [8], which seem to be very promising when it comes to inoperable ABC, the usage of Denosumab should be carefully thought through.

In conclusion, Denosumab seems to be a promising new treatment alternative in ABC, especially in patients where surgical resection is not an option and in relapsing patients where potential physical disabilities can thus be evaded.
